# Case report: Robotic repair of unroofed coronary sinus

**DOI:** 10.3389/fcvm.2022.974089

**Published:** 2022-09-28

**Authors:** Marek Pojar, Salifu Timbilla, Stepan Cerny, Mikita Karalko, Jan Vojacek

**Affiliations:** Department of Cardiac Surgery, University Hospital Hradec Kralove and Charles University, Faculty of Medicine in Hradec Kralove, Hradec Kralove, Czechia

**Keywords:** robotic surgery, unroofed coronary sinus, cardiac surgery, minimally invasive surgery, congenital heart disease

## Abstract

Unroofed coronary sinus is a rare congenital heart disease caused by the partial or complete absence of the common wall between the coronary sinus and left atrium. When indicated for repair, it is done either percutaneously or surgically. Repair using a totally endoscopic robotic procedure is rarely performed nor reported in the literature. We report a case of a 47-year-old male who underwent a successful totally endoscopic robotic repair of this anomaly.

## Introduction

Unroofed coronary sinus is a rare congenital heart disease caused by the partial or complete absence of the common wall between the coronary sinus and left atrium (1). When indicated for surgical repair, it is generally done using median sternotomy or thoracotomy. Robotic surgery has been shown to be a feasible approach to repair (2). We present a case of a successful totally endoscopic robotic repair of this anomaly in an adult using the da Vinci Xi robotic system (Intuitive Surgical, Inc., Sunnyvale, CA, United States).

## Case description

**FIGURE 1:**
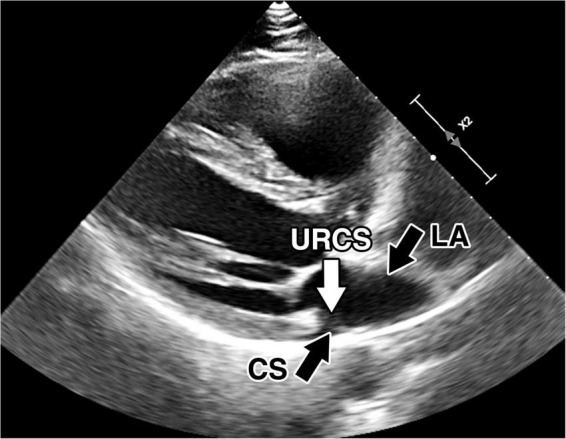
Preoperative transthoracic echocardiography. Preoperative transthoracic echocardiography images of the unroofed coronary sinus. Direct communication between the coronary sinus and left atrium. CS, coronary sinus; LA, left atrium; URCS, unroofed coronary sinus.

**FIGURE 2:**
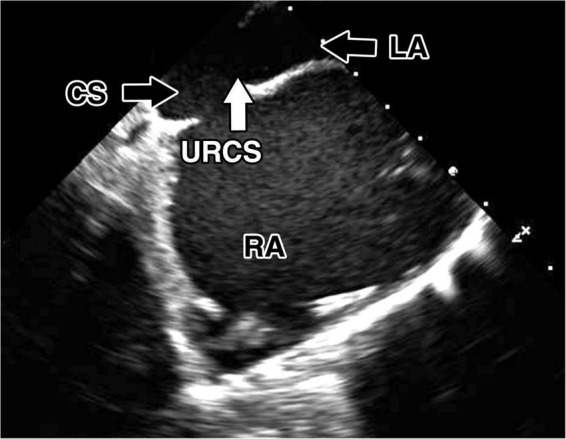
Preoperative transesophageal echocardiography. Preoperative transesophageal echocardiography images of the unroofed coronary sinus. Direct communication between the coronary sinus and left atrium. CS, coronary sinus; LA, left atrium; RA, right atrium; URCS, unroofed coronary sinus.

**FIGURE 3:**
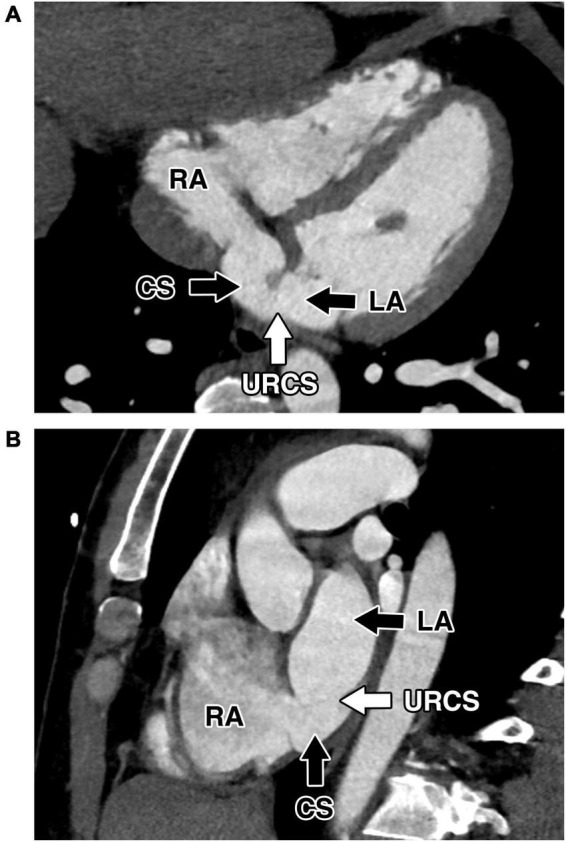
Preoperative cardiac computed tomography. Computed tomography showed extensive communication between the coronary sinus and left atrium caused by partial absence of the roof of the coronary sinus. The absence of the coronary sinus roof and communication between CS and the left atrium. **(A)** Modified four-chamber projection. **(B)** Modified short-axis projection. CS, coronary sinus; LA, left atrium; RA, right atrium; URCS, unroofed coronary sinus.

A 47-year-old male with arterial hypertension and with unroofed coronary sinus syndrome diagnosed two years before the surgery on transesophageal echocardiography (TEE) (Figures 1, 2) and cardiac computed tomography (Figures 3A,B) was referred to our clinic for repair. He presented with progression of breathlessness on exertion for the last three months. There were no significant comorbidities. Preoperative TEE revealed dilated and overloaded right ventricle, dilated right atrium and coronary sinus, and mild tricuspid regurgitation. TEE demonstrated a direct communication between the left atrium and coronary sinus with left-to-right shunt flow shown by color Doppler imaging. No atrial septal defect was seen, persistent left superior vena cava was excluded as well. Systolic pulmonary artery pressure was estimated to be 40 mmHg based on tricuspid regurgitation. Left and right ventricle function was preserved. All pulmonary veins drained into the left atrium. There was no associated heart pathology. TEE and CT visualized the defect of the roof of the coronary sinus measuring 10 mm × 12 mm. Cardiac catheterization revealed the left-to-right shunt with a Qp/Qs ratio of 1.9:1, and selective coronary angiography confirmed normal coronary arteries. The European System for Cardiac Operative Risk Evaluation (EuroScore II) was calculated to be 0.7. After careful analysis of the case and thorough discussion with the patient, we decided on a robotically assisted repair of the defect. This was due to low comorbidities and optimal anatomical proportions for a minimally invasive surgical approach. Interventional approach with an occluder was rejected due to proximity of the defect to the interatrial septum and mitral valve leaflet.

**FIGURE 4:**
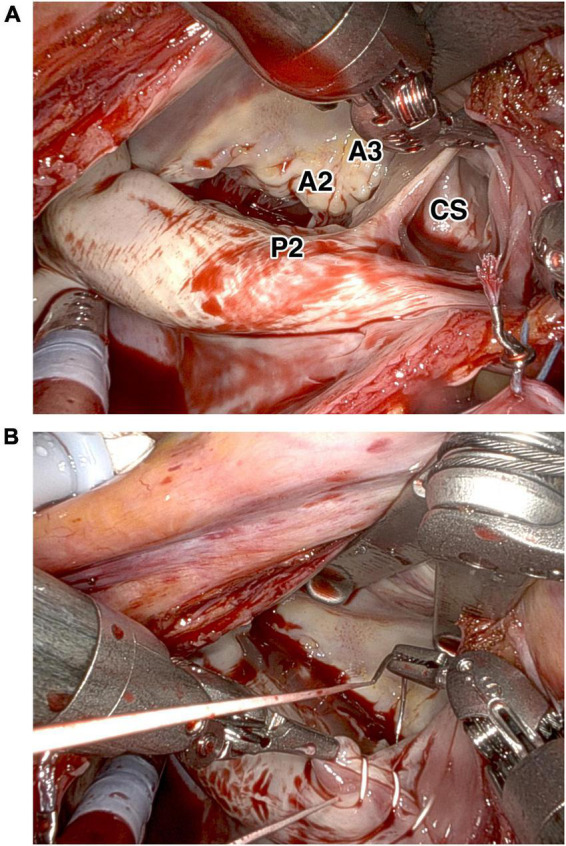
Intraoperative findings of the unroofed coronary sinus. Intraoperative findings showed **(A)** the unroofed portion of the coronary sinus at the left atrial side close to segment P3 of the posterior mitral leaflet. **(B)** Repair of the coronary sinus with direct continuous suture. A2, A2 segment of anterior mitral leaflet; A3, A3 segment of anterior mitral leaflet; P2, P2 segment of posterior mitral leaflet; CS, coronary sinus.

Under general anesthesia with double-lumen intubation and after systemic heparinization, cardiopulmonary bypass was routinely established by right internal jugular vein and right femoral vessel cannulation. The da Vinci Xi robotic surgery system (Intuitive Surgical, Inc., Sunnyvale, CA, USA) was used. Robotic ports were introduced into the right hemithorax. A 30° endoscope was inserted through the third intercostal space. Two additional instrument ports in the second and fifth intercostal space. The atrial retractor was introduced through the fifth intercostal space anteriorly. Shortly after initiating extracorporeal circulation, the ascending aorta was cross-clamped and antegrade cardioplegia (Del Nido) was used to arrest the heart in the diastole. Left atriotomy was performed. Left atrium inspection did not reveal atrial septal defect. In agreement with the TEE findings, the unroofed CS was spotted in proximity to the posterior leaflet section (P3) of the mitral valve, circular with an area of about 15 mm × 15 mm (Figures 4A,B and Supplementary Video 1). As there was sufficient tissue around the defect, we performed direct continuous suture in two layers. The atrial septum was checked for any other defects, and atrial septal defect was excluded. The CS was checked for patency. After filling the right atrium and CS, we confirmed that there was no blood cardioplegia leakage through the sutures from the repaired roof of the CS to the left atrium. This was also verified by postoperative TEE. The left atriotomy was closed, the heart de-aired, and the cross-clamp released after 57 min with spontaneous renewal of heart contractions. Perioperative TEE confirmed an excellent result of the repair with no residual defects detected. The patient was then weaned from CPB.

Patient was extubated 7 h postoperatively. After 22 h in the ICU without any complications, the patient was transferred to the standard care unit. The patient’s postoperative course was uneventful and he was discharged home 6 days after surgery without any residual shunt or new pathologies shown by postoperative transthoracic echocardiography.

## Discussion

Unroofed coronary sinus syndrome (URCS) is a rare congenital heart disease caused by the absence of part or all of the common wall between the coronary sinus and left atrium (1). Raghib et al. were the first to describe the URCS in 1965 (3). It can be classified as type I, completely unroofed with Persistent Left Superior Vena Cava; type II, completely unroofed without PLSVC; type III partially unroofed midportion of the coronary sinus (as in the current case); and type IV, partially unroofed terminal portion of the coronary sinus (4).

Clinical manifestations of URCS are variable. Symptomatology ranges from long periods of being asymptomatic to classic clinical symptoms of right-sided heart overload. URCS may cause dyspnea on exertion or at rest, fatigue, tachypnoea, right ventricular failure, or cyanosis. When URCS is associated with persistent left superior vena cava, the patient may experience central cyanosis or paradoxical embolism and brain abscess (5).

Management of URCS is dependent on the severity of symptoms and associated pathologies, such as atrial septal defect or persistent left superior vena cava, and varies from conservative therapy to acute surgery. For large defects, and also if complications related to the right-to-left shunt occur, correction of URCS becomes a necessity. In case of small, usually asymptomatic defects, only regular follow-ups are recommended (5). In addition to defect size, associated heart pathologies are important for decision making regarding intervention indication.

As imaging tools, transthoracic echocardiography is the first step to evaluate patients with suspicion of URCS. Persistent left superior vena cava could be revealed by contrast echocardiography with an injection of bubble contrast into the left arm. In the presence of persistent left superior vena cava, the contrast will appear initially in the left atrium and subsequently in the right atrium (5). Currently TEE, especially three-dimensional echocardiography, computer tomography and magnetic resonance imaging are able to confirm the presence of such abnormalities, and offer precise mapping of these structures. Prior to the therapy, cardiac catheterization and angiography have to be performed to clarify the anatomy. Special attention has to be paid to the presence of persistent left superior vena cava, a component of the Raghib syndrome, or other associated pathologies (3, 5, 6).

Treatment of URCS, if needed, involves closure of the defect causing a left-to-right shunting and correction of associated abnormalities. Classically, the treatment of URCS and persistent left superior vena cava, if needed, is surgical correction. URCS can be closed using a pericardial patch or direct sutures leaving the coronary sinus on the right and other abnormalities treated accordingly. Direct suture of the defect can be used assuming no tension is applied to the surrounding structures.

Approaches to the repair of an unroofed coronary sinus are variable. Surgical intervention is usually performed through median sternotomy. Minimally invasive approaches have been successfully employed to repair the defect. Handa et al. reported a case of a repair of a type III Unroofed coronary sinus using a right mini-thoracotomy with the aid of an endoscope (7). In their case visualization of the lesion could only be achieved with the aid of the endoscope guided by prior preoperative 3D TEE (2). Repair using robotic totally endoscopic procedure is rarely performed nor reported in the literature. Onan et al. performed and reported a successful totally endoscopic robotic repair of an unroofed CS proving the feasibility of this approach (2). Using the robotic system allows for effortless motion and better visualization. Compared to other minimally invasive approaches, robotic surgery provides quicker postoperative recovery and a better cosmetic outcome thanks to limited skin incisions. In our case, the unroofed CS was repaired successfully and safely using a total endoscopic robotic approach with an excellent postoperative outcome proving its efficacy and feasibility.

Recently, URCS can also be repaired percutaneously. Occluder devices can be employed in percutaneous procedures to close this defect with varying post-procedure complications (8). However, there are limitations for percutaneous procedure, regarding the diameter and relationship to surrounding structures.

In conclusion, unroofed coronary sinus is a rare but very important clinical problem. Robotic approach should be considered in similar cases in adult patients as it is a feasible alternative to conventional techniques.

## Data availability statement

The original contributions presented in this study are included in the article/Supplementary material, further inquiries can be directed to the corresponding author.

## Ethics statement

Ethical review and approval was not required for the study on human participants in accordance with the local legislation and institutional requirements. Written informed consent for participation was not required for this study in accordance with the national legislation and the institutional requirements. Written informed consent was not obtained from the individual(s) for the publication of any potentially identifiable images or data included in this article.

## Author contributions

MP and ST wrote the manuscript. SC and MK contributed to the conceptualization and revised the manuscript. JV performed critical revision of the manuscript and approved the final version to be published. All authors contributed to the article and approved the submitted version.

## References

[1] Kouchoukos NT Blackstone EH Hanley FL Kirklin JK. Unroofed coronary sinus syndrome. 4th ed. In: *Kirklin/Barratt-Boyes Cardiac Surgery*. Philadelphia, PA: Elsevier Sounders (2013). p. 1217–26.

[2] Onan B Aydin U Basgoze S Bakir I. Totally endoscopic robotic repair of coronary sinus atrial septal defect. *Interact Cardiovasc Thorac Surg.* (2016) 23:662–4. 10.1093/icvts/ivw200 27354465

[3] Raghib G Ruttenberg HD Anderson RC Amplatz K Adams P Jr Edwards JE. Termination of the left superior vena cava in left atrium, atrial septal defect, and absence of coronary sinus; a developmental complex. *Circulation.* (1965) 31:906–18. 10.1161/01.CIR.31.6.906 14301500

[4] Ootaki Y Yamaguchi M Yoshimura N Oka S Yoshida M Hasegawa T. Unroofed coronary sinus syndrome: diagnosis, classification, and surgical treatment. *J Thorac Cardiovasc Surg.* (2003) 126:1655–6. 10.1016/S0022-5223(03)01019-5 14666054

[5] Cinteză EE Filip C Duică G Nicolae G Nicolescu AM Bălgrădean M. Unroofed coronary sinus: update on diagnosis and treatment. *Rom J Morphol Embryol.* (2019) 60:33–40. 31263825

[6] Kawamukai M Muranaka A Yuda S Sato Y Makiguchi N Tachibana K Utility of three-dimensional transesophageal echocardiography for diagnosis of unroofed coronary sinus. *J Med Ultrason.* (2016) 43:91–4. 10.1007/s10396-015-0648-y 26703172

[7] Sandeep N Slack MC. Percutaneous management of coronary sinus atrial septal defect: two cases representing the spectrum for device closure and a review of the literature. *Cardiol Young.* (2014) 24:797–806. 10.1017/S1047951114000353 24666783

[8] Handa K Fukui S Kitahara M Kakizawa Y Nishi H. Minimally invasive surgical repair for unroofed coronary sinus syndrome directed by three-dimensional transesophageal echocardiography. *Surg Case Rep.* (2020) 6:244. 10.1186/s40792-020-00978-8 33000306 PMC7527385

